# Novel Prognostic Nomogram for Recurrence-Free Survival of Patients With Primary Gastrointestinal Stromal Tumors After Surgical Resection: Combination of Prognostic Nutritional Index and Basic Variables

**DOI:** 10.3389/fonc.2020.581855

**Published:** 2021-01-28

**Authors:** Shuliang Li, Daming Chen, Shilong Li, Zongxian Zhao, Huaxiang Yang, DaoHan Wang, Zhaoxiong Zhang, Weihua Fu

**Affiliations:** ^1^Department of General Surgery, Tianjin Medical University General Hospital, Tianjin, China; ^2^Department of Gastrointestinal Surgery, The Second People’s Hospital of Liaocheng, Linqing, China; ^3^Department of Gastrointestinal Surgery, The Second Hospital of Liaocheng Affiliated to Shandong First Medical University, Linqing, China; ^4^Department of General Surgery, Baodi People’s Hospital of Tianjin Baodi Clinical College Affiliated to Tianjin Medical University, Tianjin, China; ^5^Tianjin General Surgery Institute, Tianjin, China

**Keywords:** gastrointestinal stromal tumors, prognosis, nomogram, prognostic nutritional index, recurrence-free survival

## Abstract

**Background:**

Gastrointestinal stromal tumor (GIST) is the most common type of mesenchymal tumors in the digestive tract, often recrudescing even after R0 resection. Adjuvant tyrosine kinase inhibitor therapy prolonged recurrence-free survival (RFS). This study aimed to develop a novel nomogram for predicting the RFS of patients following surgical resection of GISTs.

**Methods:**

Clinicopathologic data of patients with GISTs at Tianjin Medical University General Hospital (Tianjin, China) from January 2000 to October 2019 were retrospectively reviewed. Univariate and multivariate Cox regression analyses were used to select the suitable variables from the training cohort to construct a nomogram for 2- and 5-year RFS. The 1,000 bootstrap samples and calibration curves were used to validate the discrimination of the nomogram. The receiver operating characteristic analysis(ROC) was used to compare the predictive ability of the nomogram and present four commonly used risk stratification systems: National Institutes of Health (NIH)–Fletcher staging system; NIH–Miettinen criteria; Modified NIH criteria; and Air Forces Institute of Pathology risk criteria (AFIP).

**Results:**

Univariate and multivariate analyses showed that the tumor site, tumor size, mitotic index, tumor rupture, and prognostic nutritional index were significant factors associated with RFS. These variables were selected to create the nomogram for 2- and 5-year RFS (all *P*<0.05). The 2- and 5-year the ROC of the nomogram were 0.821 (95% confidence interval [CI]: 0.740–0.903) and 0.798 (95% CI: 0.739–0.903); NIH–Fletcher criteria were 0.757 (95% CI: 0.667–0.846) and 0.683 (95% CI: 0.613–0.753); NIH–Miettinen criteria were 0.762 (95% CI: 0.678–0.845) and 0.718 (95% CI: 0.653–0.783); Modified NIH criteria were 0.750 (95% CI: 0.661–0.838) and 0.689 (95% CI: 0.619–0.760); and AFIP were 0.777 (95% CI: 0.685–0.869) and 0.708 (95% CI: 0.636–0.780). Hence, the predictive probabilities of our nomogram are better than those of other GIST risk stratification systems.

**Conclusion:**

This nomogram, combining tumor site, tumor size, mitotic index, tumor rupture, and prognostic nutritional index, may assist physicians in providing individualized treatment and surveillance protocols for patients with GISTs following surgical resection.

## Introduction

Gastrointestinal stromal tumors (GISTs) constitute the most common type of mesenchymal tumors in the whole digestive tract. These tumors typically arise in the gastric, and may occur in numerous sites, such as the small intestine, colon, rectum, mesentery, omentum, and retroperitoneum (1) Most GISTs originate from cajal cells, and show activation of the mutations in the KIT and platelet-derived growth factor receptor alpha (PDGFRA) proto-oncogenes (2). Complete surgical resection is the standard method for curing localized GIST. Unfortunately, these tumors often recrudesce within 5 years even after resection with negative margins (3). Some studies revealed that imatinib (a tyrosine kinase inhibitor [TKI]) may prolong recurrence-free survival (RFS) (4). The American College of Surgeons Oncology Group Trial Z9001 reported that adjuvant therapy with imatinib prolonged RFS in comparison with placebo, for patients with primary GIST≥3 cm in maximal diameter and positive for KIT protein (98% vs. 83%, respectively) (5). The National Comprehensive Cancer Network and the European Society of Medical Oncology have recommended imatinib as adjuvant treatment for patients with moderate or high risk of recurrence (6, 7). On account of the adverse effects of TKI and financial burden, it is important to precisely estimate the risk of recurrence for individual patients with GIST. Several risk stratification systems of GIST are recommended to evaluate the risk of recurrence for patients with GIST, tumor size, mitosis count, and/or tumor site, and tumor rupture: National Institutes of Health (NIH)–Fletcher staging system, NIH–Miettinen criteria, Forces Institute of Pathology (AFIP) risk criteria, and Modified NIH criteria (**Table 1**) (8–11). However, there are no generally acknowledged criteria of risk assessment for adjuvant therapy (12). Some studies presented that the neutrophil-lymphocyte ratio (NLR), platelet-lymphocyte ratio (PLR), and prognostic nutritional index (PNI) may predict recurrence and survival in patients with various solid tumors (13, 14).

**Table 1:** Commonly used risk stratification systems for GISTs.

NIH–Fletcher criteria			
**Risk**	**Feature**		
Very low	<2 cm and <5 mitotic index		
Low	2–5 cm and <5 mitotic index		
Intermediate	5–10 cm and <5 mitotic index or <5 cm and 6–10 mitotic index		
High	>5 cm and >5 mitotic index or >10 cm and any mitotic index or any size and >10 mitotic index		
**NIH**–**Miettinen criteria**			
**Group**	**Feature**		
Probably benign	Gastric: ≤5 cm and ≤5 mitotic index; Intestinal: ≤2 cm and ≤5 mitotic index		
Uncertain or low malignant potential	Gastric: >5 cm, ≤10 cm, and ≤5 mitotic index Intestinal: >2 cm, ≤5 cm, and ≤5 mitotic index		
Probably malignant	Gastric: >10 cm or >5 mitotic index; Intestinal: >5 cm or >5 mitotic index		
**AFIP–Miettinen criteria**			
**Group**	**Feature**		
Very low, if any malignant potential	≤2 cm and ≤5 mitotic index		
Low malignant potential	Gastric: >2/≤10 cm and ≤5 mitotic index, ≤2 cm and >5 mitotic index Intestinal: >2/≤5 cm and ≤5 mitotic index		
Intermediate malignant potential	Gastric: >10 cm and ≤5 mitotic index, >2/≤5 cm and >5 mitotic index Intestinal: >5/≤10 cm and ≤5 mitotic index		
High malignant potential	Gastric: >5 cm and >5 mitotic index Intestinal: >10 cm or >5 mitotic index		
**Modified NIH criteria**			
**Risk category**	**Tumor size (cm)**	**Mitotic index (per 50 HPF)**	**Location**
Very low	≤2.0	≤5.0	Any
Low	2.1–5.0	≤5.0	Any
Intermediate	≤5.0	6–10	Any
	5.1–10.0	≤5.0	Gastric
High	>10.0	Any	Any
	Any	>10	Any
	>5.0	>5	Any
	≤5.0	>5	Non-gastric
	5.1–10.0	≤5	Non-gastric

Mitotic index: number of mitoses per 50 high power fields.

Any case of tumor rapture is considered as a high-risk grade of GISTs, regardless of other factors.

AFIP, Air Forces Institute of Pathology; GIST, gastrointestinal stromal tumor; HPF, high power field; NIH, National Institutes of Health.

Nomograms are mathematical models presented by graphical illustrations, where information of certain features is used together to predict the expected endpoints. The simple graphical presentation of the nomogram allows physicians to make easy and rapid predictions in clinical practice (15). The aim of this study was to construct a nomogram that would be a practical, simple, and accurate method for predicting the RFS of patients following surgical resection of GIST, and suitable for clinical decision-making.

## Patients and Methods

A total of 525 patients diagnosed with GIST met the standard criteria at Tianjin Medical University General Hospital from January 2000 to October 2019. Finally, 392 patients who met the following criteria were included in the study (1): radical resection with negative margins; (2) availability of complete clinicopathological and follow-up data; and (3) no TKI therapy before or after surgery. The exclusion criteria were: (1) pregnancy or lactation; (2) other serious diseases or history of malignancy that may influence prognosis; (3) death within 30 days following surgery; and (4) recurrent GIST. This study was approved by the Institutional Review Board of Tianjin Medical University General Hospital.

Patient clinicopathologic data were retrospectively reviewed. The data included age, sex, tumor location, tumor size, mitotic index, treatment methods, tumor rupture results of blood testing, follow-up data, etc.

Tumor size was measured according to the maximum tumor dimensions by pathologists. Tumor sizes were categorized as: ≤2 cm; >2 cm but ≤5 cm; >5 cm but ≤10 cm); and >10 cm.

The mitotic index was determined by counting the number of mitotic figures per 50 high-power fields (HPFs) (with a breakpoint of < or ≥5 mitoses per 50 HPF). Tumor rupture was evaluated during the operation by the surgeons.

The preoperative blood test results were obtained within 7 days prior to operation. The PNI was calculated using the following formula: 10 × albumin (g/dl) + 0.005 × total number of lymphocytes. The NLR was calculated by dividing the neutrophil count by the lymphocyte count. The PLR was calculated by dividing the platelet count by the lymphocyte count. The PNI, NLR, and PLR are shown as the median (range), mean (standard deviation), and were divided into low or high group according to their median (as shown in **Table 2**).

**Table 2:** Clinicopathologic features of all patients.

Features	All patients	Feature	All patients
**Age (years)**		**Mitotic index**	
Median (range)	61.00 (19–89)	≤5 (/50 HPF)	315 (80.36%)
Mean (SD)	60.02 (11.509)	>5 (/5 HPF)	77 (19.64%)
**Sex**		**Tumor rupture**	
Male	175	Yes	32 (8.16%)
Female	217	No	360 (91.74%)
**Site**		**Hepatic metastasis**	
Gastric	254	Yes	4 (1.02%)
Non-gastric	138	No	388 (98.98%)
**Follow-up period (months)**		**PLR**	
Median (range)	32.00 (1–124)	Median (range)	107.107 (19.770–566.842)
Mean	39.57 (29.250)	Mean (SD)	122.772 (65.048)
**Tumor size (cm)**		**NLR**	
Median (range)	3.00 (0.1–32)	Median (range)	2.143 (0.076–37.496)
Mean (SD)	4.197 (4.337)	Mean (SD)	2.780 (3.142)
≤2 cm	160 (40.82%)	**PNI**	
>2 cm, ≤5 cm	133 (33.93%)	Median (range)	47.646 (26.155–65.155)
>5 cm, ≤10 cm	70 (17.86%)	Mean (SD)	46.875 (5.910)
>10 cm	29 (7.39%)		
**Recurrence**			
2-year	33 (8.42%)		
5-year	69 (17.60%)		

HPF, high power field; NLR, neutrophil-lymphocyte ratio; PLR, platelet-lymphocyte ratio; PNI, prognostic nutritional index; SD, standard deviation.

The RFS was defined as the time from complete surgical resection to the date of local recurrence, distant metastasis, or the final follow-up date. Patients who were alive without recurrence or expired without GIST recurrence were censored at the time of data collection.

The 392 patients were randomly classified into two cohorts: training cohort (n=274) and validation cohort (n=118) (ratio 7:3) (shown in **Table 3**). Univariate and multivariate Cox regression analyses were used to select the suitable variables from the training cohort to construct a nomogram for 2- and 5-year RFS.

**Table 3:** Variables of patients in the training and validation cohorts.

Variable	Training cohort (n=274)	Validation cohort (n=118)	*P^a^*	Variable	Training cohort (n=274)	Validation cohort (n=118)	*P^#^*
**Age (Years)**			0.152	**Hepatic metastasis**			0.587*
≤60	140(51.09)	51 (43.22)		Yes	2 (0.73)	2 (1.69)	
>60	134 (48.91)	67 (56.78)		No	272 (99.27)	116 (98.31)	
**Sex**			0.105	**PLR**			1.000
Male	115 (41.97)	60 (50.85)		Low(≤107.11)	137 (50.00)	59 (50.00)	
Female	159 (58.03)	58 (49.15)		High(>107.11)	137 (50.00)	59 (50.00)	
**Site**			0.558	**NLR**			0.660
Gastric	175 (63.87)	79 (66.95)		Low (≤2.143)	139 (50.73)	57 (48.31)	
Non-gastric	99 (36.13)	39 (33.05)		High (>2.143)	135 (49.27)	61 (51.69)	
**Tumor size**			0.382	**PNI**			0.509
≤2 cm	116 (42.34)	44 (37.29)		Low(≤47.646)	134 (48.91)	62 (52.54)	
>2 cm, ≤5 cm	87 (31.75)	46 (38.98)		High(>46.875)	140 (51.09)	56 (47.46)	
>5 cm, ≤10 cm	48 (17.52)	22 (18.64)		**Recurrence**			0.655
>10 cm	23 (8.39)	6 (5.08)		2-year	22 (8.03)	11 (9.32)	
**Mitotic index**			0.820	5-year	49 (17.88)	20 (16.95)	
≤5(/50 HPF)	221 (80.66)	94 (79.66)					
>5(/5 HPF)	53 (19.34)	24 (20.34)					
**Tumor rupture**			0.511				
Yes	24 (8.76)	8 (6.78)					
No	250 (91.24)	110 (93.22)					

Values are presented as number (%).

HPF, high powerfield;NLR,neutrophil-lymphocyte ratio; PLR, platelet-lymphocyte ratio; PNI, prognostic nutritional index.

**^#^**The difference between Training and Validation cohorts was calculated by chi-square test.

*Calculated by using Fisher’s exact method.

For the internal validation of the nomogram, we used the 1,000 bootstrap samples from the training cohort to obtain bias-corrected discrimination using a concordance index (c-index) (16). The c-index is a measure of the predictive accuracy for binary outcomes in a logistic regression model; values range 0.5–1.0, with higher indicating better accuracy. The calibration curves were created using the marginal estimate and the model average prediction probability; CI curves approaching the 45° line indicate better calibration. Subsequently, we used the nomogram to calculate the patients’ total scores from the validation cohort, and calculated the c-index and plotted the calibration curves to validate the discrimination again.

The receiver operating characteristic analysis was used to compare the predictive ability of the nomogram and present four commonly used risk stratification systems: NIH–Fletcher staging system; NIH–Miettinen criteria; Modified NIH criteria; and AFIP risk criteria.

For the statistical analyses, the SPSS statistics version 26.0 software (IBM Corp., Armonk, NY, USA) and R 4.0.1 software (survival and rms packages; Institute for Statistics and Mathematics, Vienna, Austria; http://www.r-project.org/) were used. A two-tailed P-value <0.05 denoted statistically significant difference.

## Results

### Clinicopathologic Features of Patients

**Tables 2** and **3** present the clinical and pathologic features of the 392 patients included in this retrospectively study. The median age at initial diagnosis was 61 years (range: 19–89 years; cutoff value: 60 years). There were 175 males and 217 females. The sites of primary tumor were gastric (n=254) and non-gastric (n=138). The median follow-up period was 32 months (range: 1–124 months). The median size of tumors was 3 cm (range: 0.1–32cm): 160 tumors ≤2 cm (40.82%), 133 tumors 2–5 cm (33.93%), 70 tumors 5–10 cm (17.86%), and 29 tumors >10 cm (7.39%). Recurrence was observed in 33 patients (8.42%) within 2 years and 69 patients (17.60%) within 5 years. There were 315 patients with mitotic index ≤ 5 per 50 HPF (80.36%) and 77 patients with mitotic index >5 per 50 HPF (19.64%). There were 32 ruptured tumors (8.16%), and four patients had hepatic metastases (1.02%). The medians and ranges of the PLR, NLR, and PNI were 107.107 (19.770–566.842), 2.143 (0.076–37.496), and 47.646 (26.155–65.155), respectively. We chose the medians of the PLR, NLR, and PNI as the cutoff points to divide the patients into low and high groups, according to a previous study (17).

### Creation of the Nomogram

**Figure 1:**
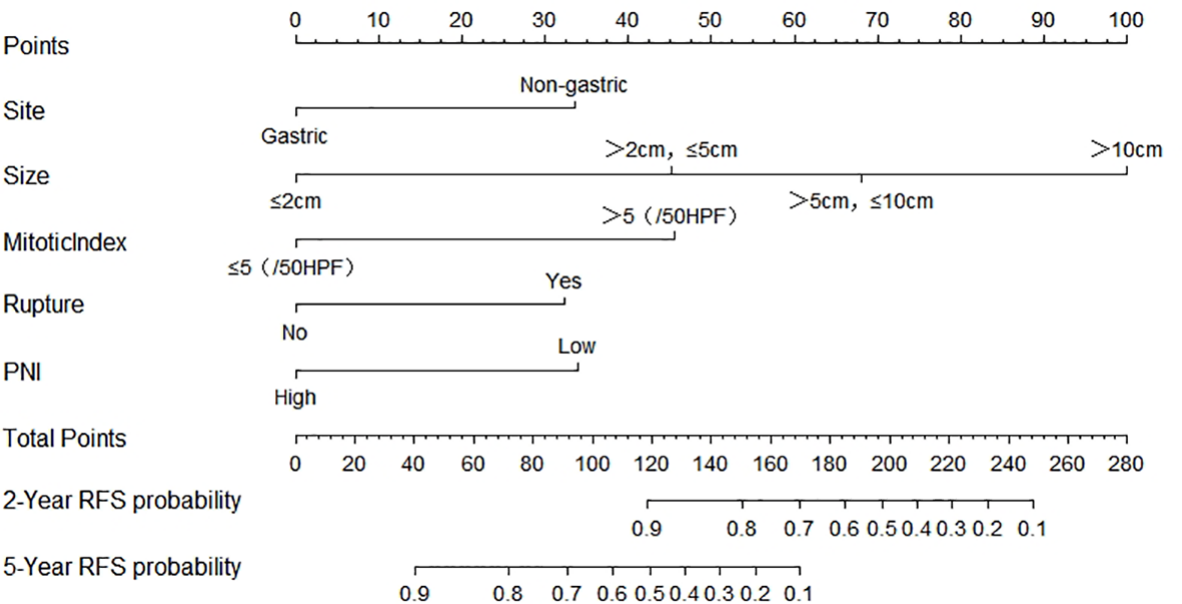
Nomogram predicting the probabilities of 2- and 5-year RFS. Points are assigned for site, size, mitotic index, rupture, and PNI by drawing a line upward from the corresponding values to the “Points” line. The sum of these five points plotted on the “Total Points” line corresponds to predictions of 2- and 5-year RFS. PNI, prognostic nutritional index; RFS, recurrence-free survival.

Firstly, this study used the univariate Cox analysis to investigate variables in the training cohort that could be significantly associated with 2- and 5-year RFS. The tumor site (hazard ratio [HR]: 4.215, 95% confidence interval [CI]: 2.235–7.950, *P*=0.000), tumor size (>2 cm and ≤5 cm, HR: 0.032, 95% CI: 0.011–0.096, *P*=0.000; >5 cm and ≤10 cm, HR: 0.122, 95% CI: 0.048–0.313, *P*=0.000; >10 cm, HR: 0.260, 95% CI: 0.101–0.666, *P*=0.005), mitotic index (HR: 5.171, 95% CI: 2.924–9.145, *P*=0.000), tumor rupture (HR: 9.489, 95% CI: 4.850–18.566, *P*=0.000), and PNI (HR: 0.266, 95% CI: 0.138–0.511, *P*=0.000) were associated with RFS (**Table 4**). Secondly, this study used the multivariate Cox analysis to select the meaningful variables. The tumor site (HR: 2.223, 95% CI: 1.129–4.377, *P*=0.000), tumor size (>2 cm and ≤5 cm, HR: 2.940, 95% CI: 1.229–7.033, *P*=0.015; >5 cm and ≤10 cm, HR: 5.059, 95% CI: 1.986–12.886, *P*=0.001; >10 cm, HR: 10.825, 95% CI: 3.208–36.531, *P*=0.000), mitotic index (HR: 2.956, 95% CI: 1.553–5.627, *P*=0.001), tumor rupture (HR: 2.163, 95% CI: 1.001–4.672, *P*=0.0496), and PNI (HR: 2.244, 95% CI: 1.091–4.614, *P*=0.028) were significant factors associated with RFS (**Table 5**, **Figure 1**).

**Table 4:** Univariate Cox analysis of prognostic variables in the training cohort for the RFS of patients with GISTs.

Variable	HR	95% CI	*P-*value	Variable	HR	95% CI	*P*-value
**Age**				**Tumor rupture**			
≤60	Ref	—	—	No	Ref	—	—
>60	0.940	0.536–1.651	0.831	Yes	9.489	4.850–18.566	0.000*
**Sex**				**Hepatic metastasis**			
Male	Ref	—	—	No	Ref	—	—
Female	1.325	0.745–2.357	0.337	Yes	0.049	0–18,709.438	0.646
**Site**				**PLR**			
Gastric	Ref	—	—	≤107.11	Ref	—	—
Non-gastric	4.215	2.235–7.950	0.000*	>107.11	1.573	0.879–2.813	0.127
**Tumor size**				**NLR**			
≤2 cm	Ref	—	—	≤2.143	Ref	—	—
>2 cm, ≤5 cm	0.032	0.011–0.096	0.000*	>2.143	1.570	0.888–2.777	0.121
>5cm, ≤10 cm	0.122	0.048–0.313	0.000*	**PNI**			
>10 cm	0.260	0.101–0.666	0.005*	≤47.646	0.266	0.138–0.511	0.000*
**Mitotic index**				>47.646	Ref	—	—
≤5 (/50 HPF)	Ref	—	—				
>5 (/5 HPF)	5.171	2.924–9.145	0.000*				

*Significant difference (P ≤ 0.05).

CI, confidence interval; GIST, gastrointestinal stromal tumor; HPF, high power field; HR, hazard ratio; NLR, neutrophil-lymphocyte ratio; PLR, platelet-lymphocyte ratio; PNI, prognostic nutritional index; RFS, recurrence-free survival; SD, standard deviation.

**Table 5:** Multivariate Cox analysis of selected prognostic variables for the RFS of patients with GISTs.

Variable	Multivariate Cox analysis		
	HR	95% CI	*P*-value
**Site**			
Gastric	Ref	—	—
Non-gastric	2.223	1.129–4.377	0.021*
**Tumor size**			
≤2 cm	Ref	—	—
>2 cm, ≤5 cm	2.940	1.229–7.033	0.015*
>5 cm, ≤10 cm	5.059	1.986–12.886	0.001*
>10 cm	10.825	3.208–36.531	0.000*
**Mitotic index**			
≤5(/50 HPF)	Ref	—	—
>5 (/5 HPF)	2.956	1.553–5.627	0.001*
**Tumor rupture**			
No	Ref	—	—
Yes	2.163	1.001–4.672	0.0496*
**PNI**			
≤47.646	2.244	1.091–4.614	0.028*
>47.646	Ref	—	—

*Significant difference (P ≤ 0.05).

CI, confidence interval; GIST, gastrointestinal stromal tumor; HPF, high power field; HR, hazard ratio; PNI, prognostic nutritional index; RFS, recurrence-free survival.

**Figure 2:**
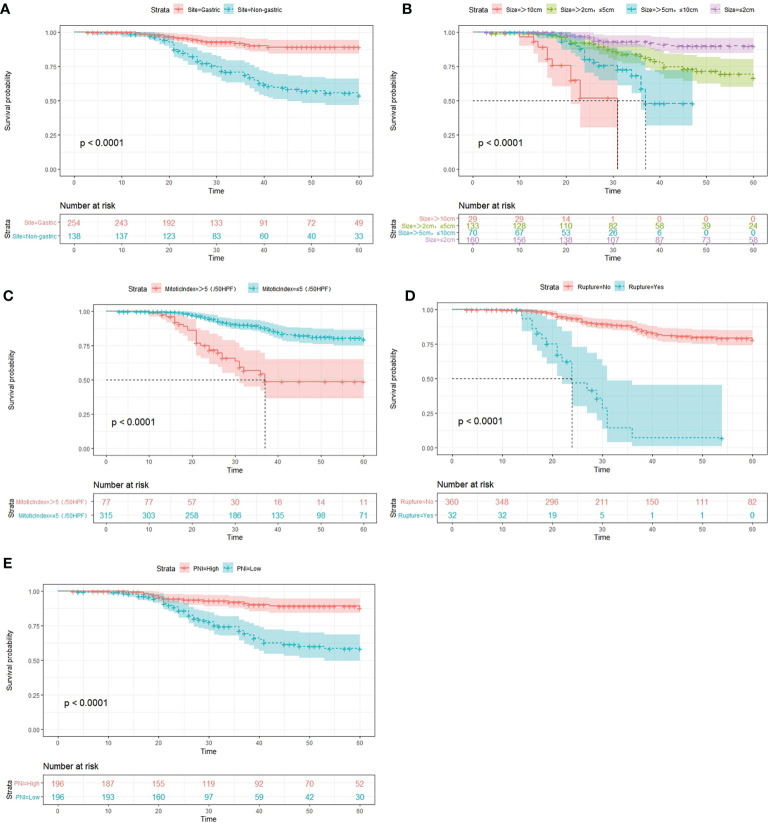
Kaplan–Meier survival curves for RFS in the whole cohort, as stratified by tumor site **(A)**, tumor size **(B)**, tumor rupture **(C)**, mitotic index **(D)**, and PNI **(E)**. PNI, prognostic nutritional index; RFS, recurrence-free survival.

Finally, based on the above results, we selected the tumor site, tumor size, mitotic index, tumor rupture, and PNI as the statistically significant predictable factors to create a nomogram for 2- and 5-year RFS (**Figure 2**). The probability of RFS at 2 and 5 years can be predicted by calculating the points of each variable and projecting the total points to the bottom scale.

### Confirmation of the Nomogram

**Figure 3:**
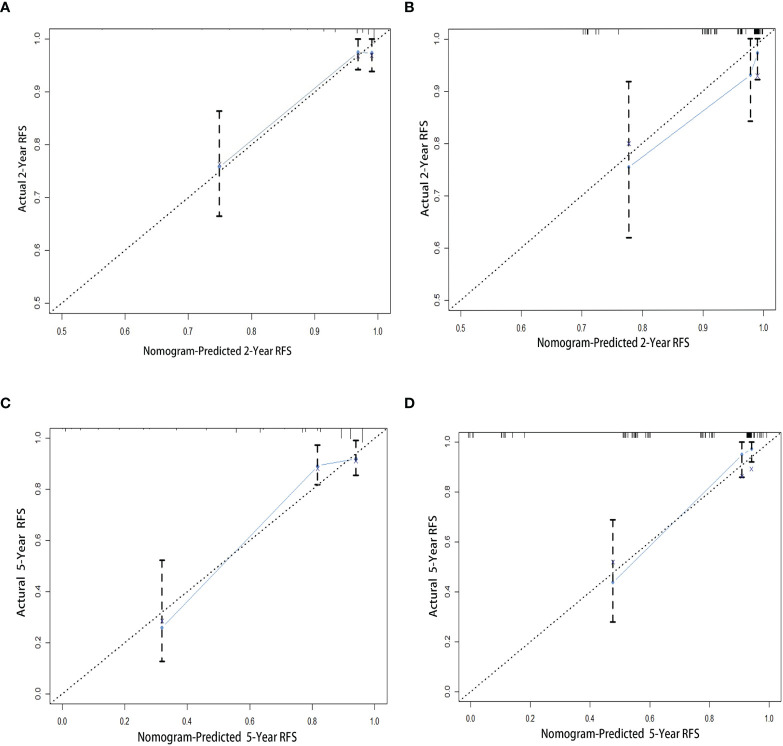
Calibration curves for the nomogram to estimate 2- and 5-year RFS in patients with GISTs. **(A)** The 2-year calibration curve in the training cohort. **(B)** The 2-year calibration curve in the validation cohort. **(C)** The 5-year calibration curve in the training cohort. **(D)** The 5-year calibration curve in the validation cohort. GIST, gastrointestinal stromal tumor; RFS, recurrence-free survival.

The 1,000 resampling bootstrap analysis was performed in the training and validation cohorts to verify the accuracy of the nomogram. The C-index of the training and validation cohorts was 0.826 (95% CI: 0.760–0.893) and 0.868 (95% CI: 0.796–0.940), respectively (**Table 6**). Meanwhile, calibration curves are illustrated in **Figure 3**, including the 2- and 5-year calibration curve in both the training and validation cohorts. Both the C-index and calibration curves in the training and validation cohorts demonstrated good consistency between the predicted and observed RFS values at 2 and 5 years. An online version of our nomogram can be accessed at https://shuliangli.shinyapps.io/DynNomapp/, to assist researchers and clinicians.

**Table 6:** C-index for nomogram predictions of RFS.

Group	C-index	95% CI
Training cohort	0.826	0.760–0.893
Validation cohort	0.868	0.796–0.940

C-index, concordance index; CI, confidence interval; RFS, recurrence-free survival.

### Comparison With Other Risk Stratification Systems

**Figure 4:**
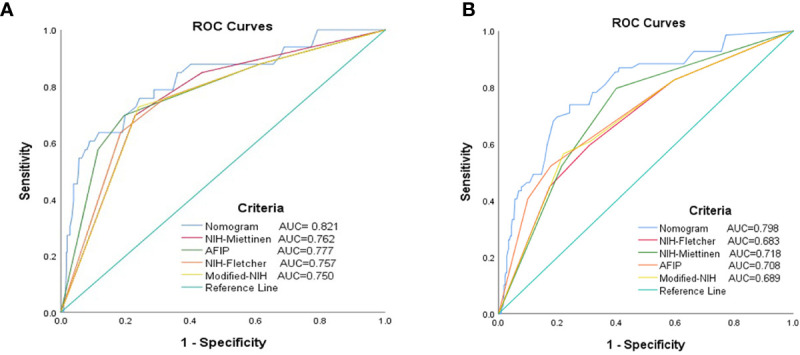
Receiver operating characteristic curves for each risk model to predict the RFS of patients with GISTs. **(A)** The 2-year ROC curves. **(B)** The 5-year ROC curves. AUC, area under the curve; GIST, gastrointestinal stromal tumor; RFS, recurrence-free survival; ROC, receiver operating characteristic.

Receiver operating characteristic analysis was performed to compare the 2- and 5-year prognostic accuracy of the nomogram and the four risk stratification systems (i.e., NIH–Fletcher, NIH–Miettinen, Modified NIH, and AFIP criteria). According to the results, the 2- and 5-year AUC were: nomogram, 0.821 (95% CI: 0.740–0.903) and 0.798 (95% CI: 0.739–0.903); NIH–Fletcher criteria, 0.757 (95% CI: 0.667–0.846) and 0.683 (95% CI: 0.613–0.753); NIH–Miettinen criteria, 0.762 (95% CI: 0.678–0.845) and 0.718 (95% CI: 0.653–0.783); Modified NIH criteria, 0.750 (95% CI: 0.661–0.838) and 0.689 (95% CI: 0.619–0.760); and AFIP, 0.777 (95% CI: 0.685–0.869) and 0.708 (95% CI: 0.636–0.780), respectively (**Table 7** and **Figure 4**). The data demonstrate that the predictive probabilities of the nomogram are better than those of other GIST risk stratification systems.

**Table 7:** The 2- and 5-year AUC according to different criteria.

Criteria	2-year AUC	2-year 95% CI	5-year AUC	5-year 95% CI
Nomogram	0.821	0.740–0.903	0.798	0.739–0.903
NIH–Fletcher	0.757	0.667–0.846	0.683	0.613–0.753
NIH–Miettinen	0.762	0.678–0.845	0.718	0.653–0.783
Modified–NIH	0.750	0.661–0.838	0.689	0.619–0.760
AFIP	0.777	0.685–0.869	0.708	0.636–0.780

*Significant difference (P ≤ 0.05).

AFIP, Air Forces Institute of Pathology; AUC, area under the curve; CI, confidence interval; NIH, National Institutes of Health.

## Discussion

At present, complete surgical resection is the primary therapeutic regimen for curing localized GISTs. However, the recurrence rate of GISTs after surgery continues to be relatively high (18, 19). In several randomized controlled trials, adjuvant TKI therapy following resection of primary GIST has been shown to increase RFS in patients at risk of recurrence (20, 21). In consideration of the adverse effects of TKI, financial burden, and patient compliance challenges, it is necessary to precisely estimate the risk of recurrence for individual patients with GIST. This would allow physicians to provide individualized treatment and surveillance protocols for such patients.

Several risk classification systems for predicting the prognosis of patients with GIST have been proposed, such as the NIH–Fletcher criteria, NIH–Miettinen criteria, AFIP risk criteria, and Modified NIH criteria. The NIH–Fletcher criteria are based on tumor size and mitotic index, while the NIH–Miettinen and AFIP criteria consider the tumor site as an additional variable. The Modified NIH criteria include the tumor size, mitotic index, tumor site, and tumor rupture as prognostic factors (11). These criteria have been compared and validated by several studies. Nevertheless, clinicians can only roughly assess the risk of recurrence after complete resection of GISTs.

The nomogram, a graphical operation interface of a mathematical model, is developed as a practical tool employing a combination of variables to precisely and rapidly predict the risk of recurrence for an individual patient. Typically, nomograms represent higher accuracy compared with other tumor staging systems (22, 23).

In 2009, utilizing the tumor size, tumor site, and mitotic index, Gold et al. created a prognostic nomogram to predict the RFS for patients with localized primary GISTs after complete surgical resection. It was termed the Memorial Sloan Kettering Cancer Center (MSKCC) nomogram (24). Some studies were employed to validate the prognostic predictive probability of the MSKCC nomogram through comparison with other risk classification criteria. The findings revealed that the prediction performance of the MSKCC nomogram for RFS was better than that of the NIH criteria; however, there was no significant difference between the MSKCC nomogram and the AFIP criteria (24, 25).

Some researchers consider that it is possible to improve the risk predictive probability of current risk stratification criteria by adding reasonable variables (25, 26). To identify additional predictive factors besides tumor site, size, mitotic index, and tumor rupture, this study retrospectively analyzed the clinicopathologic data of patients with GISTs through univariate and multivariate Cox analyses. The results showed that the PNI may be a predictive factor. The nomogram was constructed by combining the tumor site, size, mitotic index, tumor rupture, and PNI. It demonstrated excellent performance compared with other risk stratification criteria. To the best of our knowledge, this nomogram is the first to include the PNI as a predictive factor.

Tumor site, size, and mitotic index have been widely utilized to establish several risk stratifications. Consistent with the present results, several studies have validated their significance as prognostic factors (8–10, 23, 27).

Similar to previous studies (23–26), in our study, we found that the recurrence risk of non-gastric GISTs is higher than that of gastric GISTs.

Tumor size was transferred from a continuous variable to categorical variable, and divided into four groups for analysis. We found that it is the dominant risk factor of recurrence, according to the multivariate Cox analysis.

Mitotic index is another risk factor; in this study, patients with a mitotic index >5/50 HPFs had shorter RFS than those with a mitotic index <5/50 HPFs.

Tumor size and the mitotic index are important for evaluating the RFS of patients with localized primary GISTs after complete surgical resection. However, there are some factors influencing their accuracy, such as the fixed time after resection, the quality of pathological section, the pathologist’s experience, and the selected areas for counting mitotic and different microscopes used by the pathologists (12). For instance, there are two different microscopes. For the older one used in AFIP studies, the area of per 50 HPF is 5.3 mm^2^; however, for the currently used wide-field microscope, the area of 50 HPF is 11.87 mm^2^, the difference between the two microscopes is approximately 2.2-fold. In addition, some researchers counted mitoses in the most mitotically active area or the most cellular area, or until >100 mitoses were found, others counted in 50 randomly selected HPFs (28). To reduce the influence, pathologists could choose the same microscope and count per total area of 5 mm^2^ (12).

Tumor rupture is an independent prognostic factor, and its incidence ranges 2%–22% (13, 21). Some studies exhibited that GISTs patients without tumor rupture had a significantly longer RFS after complete resection than those with tumor rupture (11, 28). Joensuu et al. included tumor rupture as a risk factor in the Modified NIH criteria (11). In this study, the rate of tumor rupture in the cohort was 8.16%. Meanwhile, the Cox analysis showed that it was a significant predictive variable, consistent with previous studies.

In recent years, several findings have demonstrated that inflammatory parameters may be perfect factors as prognostic markers of various malignancies, including the NLR, PLR, and PNI (29–31). All three markers are easily acquired from complete blood counts and liver function testing. These tests are routinely used in clinical practice, and will not incur additional financial burden for patients.

Unfortunately, the present findings [similar to that of Shi et al (32).] revealed that only the PNI was an independent prognostic factor in patients with GISTs. The PNI, composed of albumin and lymphocytes, was initially used to evaluate the immunological and nutritional aspects of patients undergoing gastrointestinal tract surgery (29). Numerous previous studies have shown that low PNI was associated with poorer progression-free survival and overall survival in patients with malignant tumors, including high recurrence risk for GISTs (17, 30, 32, 33). In this study, the results indicated that GIST patients with a low PNI had shorter RFS than those with a high PNI.

Low PNI is also associated with hypoalbuminemia and/or lymphocytopenia. Hypoalbuminemia is an inflammatory surrogate rather than nutritional marker among these patients with cancer, and serum albumin reflects the inflammatory and nutritional status of patients (33). The bodies of patients with cancer undergo physiological stress, such as tumor hypoxia/necrosis and local tissue damage caused by cancer cells. To counteract these stimuli, the immune system of the body responds with a systemic release of proinflammatory cytokines and growth factors. Meanwhile, the hepatic cells promote the production of acute-phase proteins, and reduce the production of albumin, especially among patients with advanced cancer (34). Besides, GISTs may cause some symptoms, such as digestive bleeding, dyspepsia, nausea, vomiting, and diarrhea, which may result in malnutrition and hypoalbuminemia.

Another essential factor of the PNI is lymphocytes, that play an important role in enhancing the host immune system and eliminate the proliferation, migration, and invasion of cancer cells (35). Thus, patients with tumors and lymphocytopenia have impaired cellular immune systems and are unable to produce appropriate inflammatory reaction (36, 37). Wu et al. thought that patients who can maintain lymphocyte counts may have a reboot immune reaction, which might convey an optimistic prognosis in cancer (38).

As demonstrated in **Figure 3**, the nomogram has a satisfactory predictable performance compared with other risk stratification systems, with 2- and 5-year values of 0.821 and 0.798, respectively. Although the MSKCC nomogram was not included in the present analysis, Gold et al. reported that the MSKCC nomogram performs similarly to the AFIP criteria (c-index: 0.76; *P*=0.33) (24).

This study had several limitations. Firstly, this was a retrospective study, and may include biases in the analyses. Thus, prospective cohort studies are warranted to validate our conclusions. Secondly, this study was conducted in a single center. Hence, multi-center validation in the future is necessary to verify the importance of this nomogram.

## Conclusions

In the present study, a nomogram based on tumor site, tumor size, mitotic index, tumor rupture, and the PNI, was constructed to predict the RFS of patients with primary GISTs. This nomogram may assist physicians in providing individualized treatment and surveillance protocols for patients with GISTs.

## Data Availability Statement

The raw data supporting the conclusions of this article will be made available by the authors, without undue reservation.

## Ethics Statement

The studies involving human participants were reviewed and approved by Institutional Review Board of Tianjin Medical University General Hospital. Written informed consent for participation was not required for this study in accordance with the national legislation and the institutional requirements.

## Author Contributions

ShuL prepared the manuscript and performed the statistical analysis. WF presented the concept of the study and designed the study. DC reviewed the manuscript. ShiL, ZoZ, and DW contributed to the data acquisition, analysis, and interpretation. HY and ZhZ performed the data analysis. All authors contributed to the article and approved the submitted version.

## Conflict of Interest

The authors declare that the research was conducted in the absence of any commercial or financial relationships that could be construed as a potential conflict of interest.

## References

[1] Miettinen M Lasota J Gastrointestinal stromal tumors (GISTs): definition, occurrence, pathology, differential diagnosis and molecular genetics. Pol J Pathol (2003) 54(1):3–24. 10.1007/s004280000338 12817876

[2] Daniels M Lurkin I Pauli R Erbstösser E Hildebrandt U Hellwig K Spectrum of KIT/PDGFRA/BRAF mutations and phosphatidylinositol-3-kinase pathway gene alterations in gastrointestinal stromal tumors (GIST). Cancer Lett (2011) 312:43–54. 10.1016/j.canlet.2011.07.029 21906875

[3] Dematteo RP Ballman KV Antonescu CR Maki RG Pisters PW Demetri GD Adjuvant imatinib mesylate after resection of localised, primary gastrointestinal stromal tumour: a randomised, double blind, placebo-controlled trial. Lancet (2009) 373:1097–104. 10.1016/S0140-6736(09)60500-6 PMC2915459 19303137

[4] Poveda A Garcia Del Muro X Lopez-Guerrero JA Cubedo R Martínez V Romero I GEIS guidelines for gastrointestinal sarcomas (GIST). Cancer Treat Rev (2017) 55:107. 10.1016/j.ctrv.2016.11.011 28351781

[5] Dematteo R Adjuvant imatinib mesylate increases recurrence free survival (RFS) in patients with completely resected localized primary gastrointestinal stromal tumor (GIST): North American Intergroup Phase III trial ACOSOG. Asco Meeting;Chicago, IL, USA (2007).

[6] National Comprehensive Cancer Network (NCCN) NCCN clinical practice guidelines in oncology. J Natl Compr Canc Netw (2016) 14(6):758–86. 10.6004/jnccn.2016.0078 27283169

[7] ESMO/European Sarcoma Network Working Group Gastrointestinal stromal tumours: ESMO Clinical Practice Guidelines for diagnosis, treatment and follow-up. Ann Oncol (2014) 25(Suppl 3):iii21–26. 10.1093/annonc/mdu255 25210085

[8] Fletcher CD Berman JJ Corless C Gorstein F Lasota J Longley BJ Diagnosis of gastrointestinal stromal tumors: A consensus approach. Hum Pathol (2002) 33:459–65. 10.1053/hupa.2002.123545 12094370

[9] Miettinen M El-Rifai W Sobin LHL Lasota J Evaluation of malignancy and prognosis of gastrointestinal stromal tumors: a review. Hum Pathol (2002) 33:478–83. 10.1053/hupa.2002.124123 12094372

[10] Miettinen M Lasota J Gastrointestinal stromal tumors: review on morphology, molecular pathology, prognosis, and differential diagnosis. Arch Pathol Lab Med (2006) 130:1466–78. 10.1043/1543-2165(2006)130[1466:GSTROM]2.0.CO;2 17090188

[11] Joensuu H Risk stratification of patients diagnosed with gastrointestinal stromal tumor. Hum Pathol (2008) 39:1411–9. 10.1016/j.humpath.2008.06.025 18774375

[12] Chen T Xu L Ye L Yu J A new nomogram for recurrence-free survival prediction of gastrointestinal stromal tumors: Comparison with current risk classification methods. Eur J Surg Oncol (2019) 45(6):1109–14. 10.1016/j.ejso.2018.12.014 30594406

[13] Okada S Shimada J Kato D Tsunezuka H Teramukai S Inoue M Clinical significance of prognostic nutritional index after surgical treatment in lung cancer. Ann Thorac Surg (2017) 104:296–302. 10.1016/j.athoracsur.2017.01.085 28433217

[14] Stotz M Liegl-Atzwanger B Posch F Mrsic E Thalhammer M Stojakovic T Blood-based biomarkers are associated with disease recurrence and survival in gastrointestinal stroma tumor patients after surgical resection. PloS One (2016) 11(7):e0159448. 10.1371/journal.pone.0159448 27454486 PMC4959723

[15] Balachandran VP Gonen M Smith JJ DeMatteo RP Nomograms in oncology: more than meets the eye. Lancet Oncol (2015) 16(4):e173–80. 10.1016/S1470-2045(14)71116-7 PMC4465353 25846097

[16] Harrell FE Jr Califf RM Pryor DB Lee KL Rosati RA Evaluating the yield of medical tests. JAMA (1982) 247(18):2543–6. 7069920

[17] Shi WK Zhang XH Zhang J Wei ZW Predictive ability of prognostic nutritional index in surgically resected gastrointestinal stromal tumors: a propensity score matching analysis. Jpn J Clin Oncol (2019) 49(9):823–31. 10.1093/jjco/hyz078 31162583

[18] Joensuu H Vehtari A Riihimaki J Nishida T Steigen SE Brabec P Risk of recurrence of gastrointestinal stromal tumour after surgery: an analysis of pooled population-based cohorts. Lancet Oncol (2012) 13:265–74. 10.1016/S1470-2045(11)70299-6 22153892

[19] Keung EZ Raut CP Management of gastrointestinal stromal tumors. Surg Clin North Am (2017) 97(2):437–52. 10.1016/j.suc.2016.12.001 28325196

[20] Nishida T Blay JY Hirota S Kang YK The standard diagnosis, treatment, and follow-up of gastrointestinal stromal tumors based on guidelines. Gastric Cancer (2016) 19(1):3–14. 10.1007/s10120-015-0526-8 26276366 PMC4688306

[21] Joensuu H Eriksson M Sundby Hall K Reichardt P One vs three years of adjuvant imatinib for operable gastrointestinal stromal tumor: a randomized trial. JAMA (2012) 307(12):1265–72. 10.1001/jama.2012.347 22453568

[22] Wang ZX Qiu MZ Jiang YM Xu RH Comparison of prognostic nomograms based on different nodal staging systems in patients with resected gastric cancer. J Cancer (2017) 8(6):950–8. 10.7150/jca.17370 PMC5436246 28529606

[23] Chen Z Lin RM Bai YK Zhang Y Establishment and verification of prognostic nomograms for patients with gastrointestinal stroma l tumors: A SEER-based study. BioMed Res Int (2019) 2019:8293261. 10.1155/2019/8293261 31032364 PMC6457297

[24] Gold JS Gönen M Gutiérrez A DeMatteo RP Development and validation of a prognostic nomogram for recurrence-free survival after complete surgical resection of localised primary gastrointestinal stromal tumour: a retrospective analysis. Lancet Oncol (2009) 10(11):1045–52. 10.1016/S1470-2045(09)70242-6 PMC3175638 19793678

[25] Belfiori G Sartelli M Cardinali L Marmorale C Risk stratification systems for surgically treated localized primary Gastrointestinal Stromal Tumors (GIST). Review of literature and comparison of the three prognostic criteria: MSKCC Nomogram, NIH–Fletcher and AFIP–Miettinen. Ann Ital Chir (2015) 86(3):219–27. 10.1016/S1470-2045(09)70242-6 26098671

[26] Racz JM Brar SS Cleghorn MC Jimenez MC Azin A Atenafu EG The accuracy of three predictive models in the evaluation of recurrence rates for gastrointestinal stromal tumours. J Surg Oncol. (2015) 111(4):371–6. 10.1002/jso.23839 25501790

[27] Goh BK Chow PK Yap WM Kesavan SM Song IC Paul PG Which is the optimal risk stratification system for surgically treated localized primary GIST? Comparison of three contemporary prognostic criteria in 171 tumors and a proposal for a modified Armed Forces Institute of Pathology risk criteria. Ann Surg Oncol (2008) 15:2153–63. 10.1245/s10434-008-9969-z 18546045

[28] Dematteo RP Gold JS Saran L Antonescu CR Tumor mitotic rate, size, and location independently predict recurrence after resection of primary gastrointestinal stromal tumor (GIST). Cancer (2008) 112(3):608–15. 10.1002/cncr.23199 18076015

[29] Chan AW Chan SL Wong GL Wong VW Chong CC Lai PB Prognostic nutritional index (PNI) predicts tumor recurrence of very early/early stage hepatocellular carcinoma after surgical resection. Ann Surg Oncol (2015) 22(13):4138–48. 10.1245/s10434-015-4516-1 25801356

[30] Geng Y Shao Y Zhu D Zheng X Zhou Q Zhou W Systemic immune-inflammation index predicts prognosis of patients with esophageal squamous cell carcinoma: A propensity score-matched analysis. Sci Rep (2016) 6:39482. 10.1038/srep39482 28000729 PMC5175190

[31] Yilmaz A Mirili C Bilici M Tekin SB A novel predictor in patients with gastrointestinal stromal tumors: Systemic immune-inflammation index (SII). J BUON (2019) 24(5):2127–35. 31786885

[32] Cadwell JB Afonso AM Shahrokni A Prognostic nutritional index (PNI), independent of frailty is associated with six-month postoperative mortality. J Geriatr Oncol (2020) 11(5):880–4. 10.1016/j.jgo.2020.03.013 PMC8311543 32253157

[33] Sun J Mei Y Zhu Q Shou C Tjhoi WEH Yang W Relationship of prognostic nutritional index with prognosis of gastrointestinal stromal tumors. J Cancer (2019) 10(12):2679–86. 10.7150/jca.32299 PMC6584938 31258776

[34] Lis CG Grutsch JF Vashi PG Lammersfeld CA Is serum albumin an independent predictor of survival in patients with breast cancer? J Parenter Enteral Nutr (2003) 27:10–5. 10.1177/014860710302700110 12549592

[35] Nazha B Moussaly E Zaarour M Azab B Hypoalbuminemia in colorectal cancer prognosis: Nutritional marker or inflammatory surrogate? World J Gastrointest Surg (2015) 7(12):370–7. 10.4240/wjgs.v7.i12.370 PMC4691717 26730282

[36] Mirili C Yılmaz A Demirkan S Basol Tekin S Clinical significance of prognostic nutritional index (PNI) in malignant melanoma. Int J Clin Oncol (2019) 24(10):1301–10. 10.1007/s10147-019-01461-7 31073814

[37] Caputo F Dadduzio V Tovoli F Bertolini G Cabibbo G Cerma K The role of PNI to predict survival in advanced hepatocellular carcinoma treated with sorafenib. PloS One (2020) 15(5):e0232449. 10.1371/journal.pone.0232449 32379785 PMC7205300

[38] Wu ES Oduyebo T Cobb LP Cholakian D Kong X Fader AN Lymphopenia and its association with survival in patients with locally advanced cervical cancer. Gynecol Oncol (2016) 140(1):76–82. 10.1016/j.ygyno.2015.11.013 26571200 PMC4782779

